# Genetic variants in systemic lupus erythematosus susceptibility loci, *XKR6* and *GLT1D1* are associated with childhood-onset SLE in a Korean cohort

**DOI:** 10.1038/s41598-018-28128-z

**Published:** 2018-07-02

**Authors:** Young Bin Joo, Jiwoo Lim, Betty P. Tsao, Swapan K. Nath, Kwangwoo Kim, Sang-Cheol Bae

**Affiliations:** 1 Department of Rheumatology, St. Vincent’s Hospital, The Catholic University of Korea, Suwon, Republic of Korea; 2 Department of Biology, Kyung Hee University, Seoul, Republic of Korea; 3 Division of Rheumatology and Immunology, Department of Medicine, Medical University of South Carolina, Charleston, South Carolina USA; 4 Arthritis and Clinical Immunology Research Program, Oklahoma Medical Research Foundation, Oklahoma City, Oklahoma USA; 5 Department of Rheumatology, Hanyang University Hospital for Rheumatic Diseases, Seoul, Republic of Korea

## Abstract

Impact of genetic variants on the age of systemic lupus erythematosus (SLE) onset was not fully understood. We investigated a cumulative effect of SLE-risk variants on the age of SLE onset and scanned genome-wide SNPs to search for new risk loci of childhood-onset SLE (cSLE). We analyzed 781 Korean single-center SLE subjects who previously genotyped by both Immunochip and genome-wide SNP arrays. Individual genetic risk scores (GRS) from well-validated SLE susceptibility loci were calculated and tested for their association with cSLE (<16 years at onset). Single-variant association tests were performed using a multivariable logistic regression adjusting for population stratification. GRS from SLE susceptibility loci was significantly higher in cSLE than aSLE (p = 1.23 × 10^−3^). Two SNPs, rs7460469 in *XKR6* (p = 1.26 × 10^−8^, OR = 5.58) and rs7300146 in *GLT1D1* p = 1.49 × 10^−8^, OR = 2.85), showed the most significant associations with cSLE. The model consisting of GRS of SLE and two newly identified loci showed an area under curve (AUC) of 0.71 in a receiver operating characteristics (ROC) curve for prediction of cSLE. In conclusion, cSLE is associated with a high cumulative SLE-risk effect and two novel SNPs rs7460469 and rs7300146, providing the first predictive model for cSLE in Koreans.

## Introduction

Systemic lupus erythematosus (SLE) is a polygenic autoimmune disease representing mild to severe manifestations. SLE could occur in all ages, but it has been known that patients with childhood-onset SLE (cSLE) have worse clinical outcomes than those with adult-onset SLE (aSLE). For examples, severe clinical features such as lupus nephritis, neuropsychiatric SLE, and hemolytic anemia were more frequent in cSLE and higher mortality risk was also observed in cSLE in the comparison with aSLE^1,2^.

There is incomplete explanation about the difference in the age of onset and the prognosis between cSLE and aSLE. It has been thought that genetic differences might affect the age of SLE onset. Nevertheless, no systematic genetic approaches like a genome-wide association study to identify relevant variants or genes have been perform. Instead, because susceptibility loci are related with disease development, SLE-risk variants have been investigated for their cumulative effects on the age of SLE onset. So far, there have been two studies on this view. Taylor *et al*. showed that cumulative genetic risk score (GRS) calculated from the effect size and number of SLE-risk alleles is associated with age at diagnosis of SLE^3^. However, early and late onset of SLE was defined as before and after 34 years of age, respectively, which did not reflect cSLE and aSLE. Webb *et al*. also identified that the number of SLE-risk alleles was more in patients with cSLE^4^. However, the relatively different effect sizes among the risk variants were ignored. The observed association was marginal and not confirmed in the European population, the largest ancestral group in that study. Additionally, both the studies used HLA SNPs that are hard to explain majority of the SLE associations in the HLA region and they calculated individual’s genetic burdens from the small numbers of SLE loci found at the time of publications.

Here, we investigated the genetic contribution on cSLE, overcoming the limitations of the previous researches. A cumulative SLE-risk effect in an individual from the current HLA amino-acid haplotypes and the latest non-HLA SLE-risk variants was calculated and tested for the association with cSLE (diagnosed before the age of 16). We also searched for new risk loci of cSLE using genome-wide SNP data in a Korean population.

## Results

### Characteristics of cSLE and aSLE

Mean age of SLE onset was 12.5 ± 2.5 years in 96 cSLE patients and 29.0 ± 9.4 in 685 aSLE patients. cSLE showed worse clinical outcomes regarding to SLE specific manifestations and disease activity. The number of cumulative American College of Rheumatology (ACR) criteria and the adjusted mean SLE disease activity index (AMS) were higher in cSLE than aSLE during follow-up period (p < 0.05, Table 1).

**Table 1:** Baseline and cumulative clinical characteristics of cSLE and aSLE.

	cSLE	aSLE	p
Age of SLE onset, year, mean ± SD	12.5 ± 2.5	29.0 ± 9.4	<0.001
Sex, female, N (%)	78 (81.3)	653 (95.3)	<0.001
Disease duration, year, mean ± SD	3.3 ± 3.6	2.9 ± 3.4	0.248
Follow-up duration, year, mean ± SD	10.7 ± 4.7	10.5 ± 5.3	0.770
range	1.7–20.9	0.2–30.3	
At the time of enrollment			
The number of ACR criteria, mean ± SD	5.3 ± 1.4	5.1 ± 1.4	0.210
SLEDAI, mean ± SD	5.6 ± 4.6	5.5 ± 4.4	0.897
SDI > 1, N (%)	19 (19.8)	204 (29.8)	0.42
At the time of last follow-up			
The number of ACR criteria, mean ± SD	6.1 ± 1.6	5.6 ± 1.4	0.001
AMS, mean ± SD	4.9 ± 2.6	4.2 ± 2.6	0.017
SDI > 1, N (%)	38 (39.6)	325 (47.7)	0.148

cSLE, childhood-onset systemic lupus erythematosus; aSLE, adult-onset systemic lupus erythematosus; SD, standard deviation; ACR, American College of Rheumatology; SLEDAI, the SLE disease activity index; SDI, the Systemic Lupus International Collaborating Clinics/ACR SLE Damage Index; AMS, the Adjusted Mean SLEDAI-2K.

### Association of genetic risk score with cSLE

**Figure 1:**
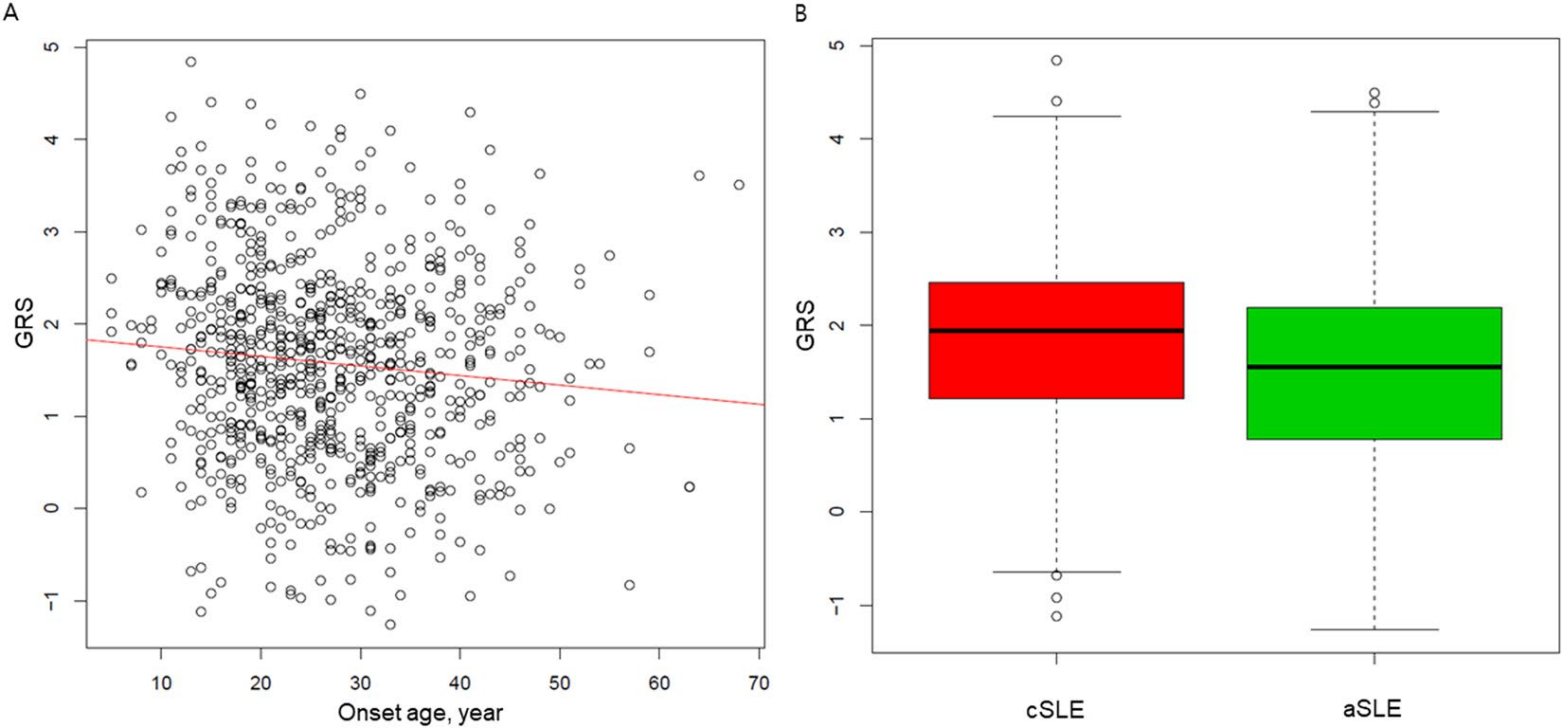
Distribution of weighted GRS (**A**) according to the age of SLE onset and (**B**) between cSLE and aSLE. cSLE; childhood-onset SLE, aSLE; adult-onset SLE, GRS; genetic risk score.

Weighted GRS calculated from non-HLA and HLA SLE-risk loci was ranged from −1.26 to 4.83 (median GRS = 1.58, first quartile = 0.81, third quartile = 2.25) showed significantly negative correlation with the age of SLE onset in a linear regression model (p = 1.23 × 10^−3^). With every one year increase in the age of onset, the weighted GRS was decreased by about 0.036 (*β* coefficient = −0.036, 95% CI = −0.047 to −0.025; Fig. 1A). Consistently, a significant difference in the weighted GRS between cSLE and aSLE was observed in logistic regression model (median GRS = 1.94 in cSLE and 1.55 in aSLE; p = 6.69 × 10^−3^; OR = 2.59, 95% CI = 1.82–3.37) (Fig. 1B).

### Association of rs7460469 and rs7300146 with cSLE

**Figure 2:**
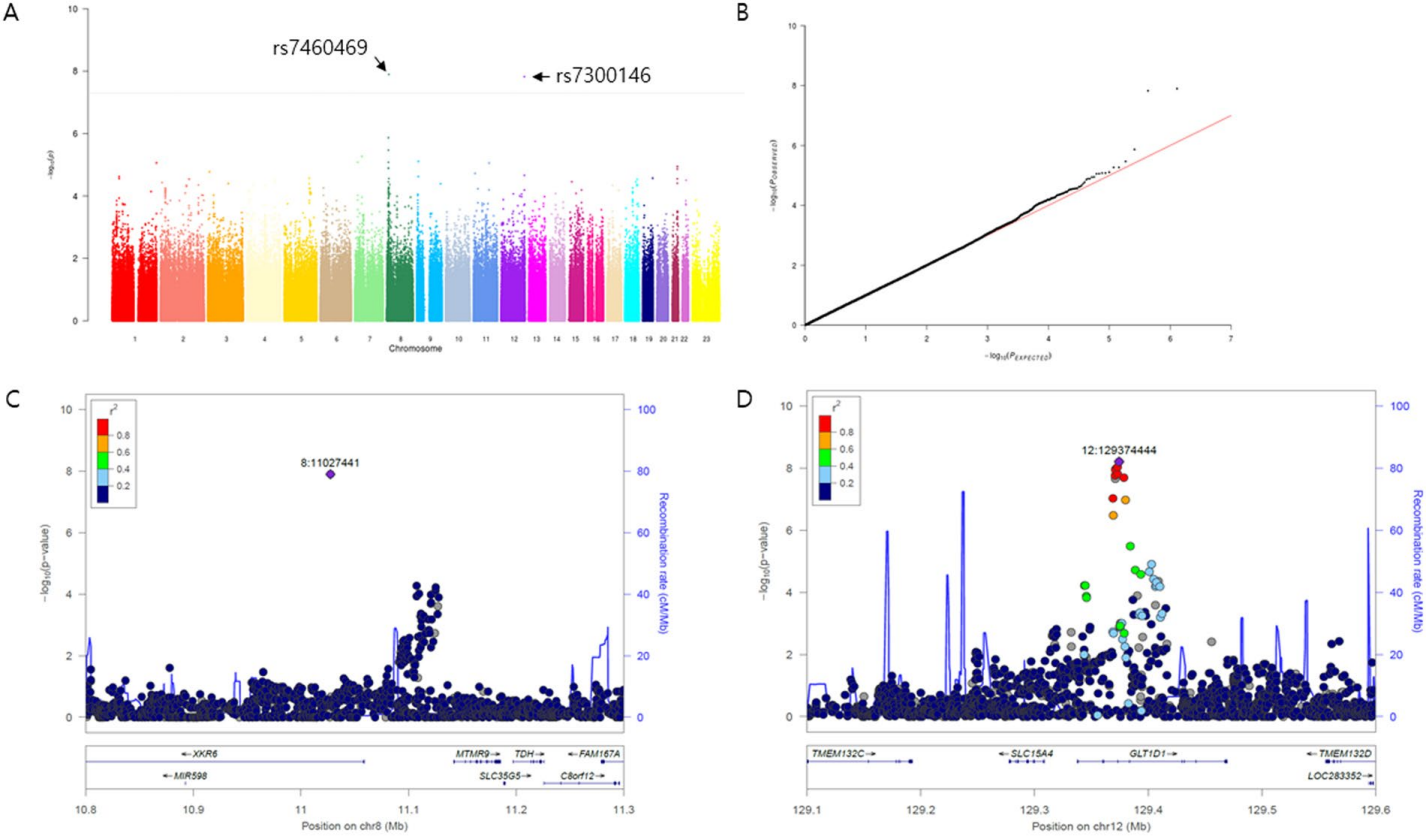
Logistic regression analysis was performed to identify genetic variants associated with cSLE. (**A**) Manhattan plot for the genome-wide association results of c SLE. The negative log_10_ of association p value (y-axis) of each variant is plotted according to its chromosomal position (**B**) Quantile–quantile plot showing the observed p values against the expected distribution under the null hypothesis. (**C**) Regional association plot for rs7460469 in *XKR6* after imputation. (**D**) Regional association plot for rs7300146 in *GLT1D1* after imputation. cSLE; childhood-onset SLE.

A total of 648,077 genome-wide SNPs were obtained with the average call rate of 99.7%, after the quality control procedure. We identified two loci associated with cSLE that surpassed the genome-wide significance level in multivariable logistic regression model (Table 2 and Fig. 2A), with the genomic inflation factor of 1.003 (Fig. 2B). The most significant signal (p = 1.26 × 10^−8^, OR = 5.58) was at the genotyped SNP rs7460469 in *XKR6* (Fig. 2C). Notably, this locus was located between *FAM167A* (previously referred to as *C8orf13*) and *BLK*, which were demonstrated as an SLE risk loci in Asians as well as Caucasians^5–8^. The SNP rs7460469 was polymorphic only in Asian populations according to the 1000 Genomes Project data. The SLE-risk variants reported in *FAM167A-BLK* (rs922483 and rs1382568)^5^ were not correlated with rs7460469 in *XKR6* and showed no association with cSLE in the study subjects (p > 0.05).

**Table 2:** Significant associations of two genotyped SNPs with cSLE in Korean cohort (n = 781).

Chr	SNP	Major allele	Minor allele	MAF in aSLE	MAF in cSLE	OR for minor allele	95% CI	p
8	rs7460469	G	A	0.03	0.11	5.58	3.09–10.10	1.26 × 10^−8^
12	rs7300146	A	C	0.17	0.35	2.85	1.98–4.10	1.49 × 10^−8^

OR; odds ratio, CI; confidence interval.

We note that the local association was supported by only a single SNP rs7460469 without its correlated SNPs with similar association *P* values. In order to ensure the association of rs7460469, we checked that the raw fluorescence signals in the rs7460469 assays clearly cluster into three discrete genotypic groups (data not shown) and the distribution of genotypes in the subjects follows Hardy-Weinberg equilibrium (HWE; P_HWE_ = 1.00). In addition, we re-genotyped the same variant in a subset (n = 110 with 1 *AA*, 56 *AG* and 53 *GG*) of the study subjects using the Sanger sequencing method. The genotype results between GWAS array and sequencing were concordant 100% in the 110 samples. Linkage disequilibrium (LD) between rs7460469 and its neighboring SNPs was also calculated, based on the Asian LD information in the 1000 Genomes Projects database. There was no SNP in LD (r^2^ > 0.2) with rs7460469, which supports the observed regional association plot with only an associated variant (Fig. 2C).

The other significant signal (p = 1.49 × 10^−8^, OR = 2.84) was detected at rs7300146 in *GLT1D1* (Fig. 2D). High genotyping quality for rs7300146 was assessed by checking the raw fluorescence signals in the genotyping assays (data not shown). We re-genotyped the same variant in the subset (n = 110 with 24 *CC*, 57 *AG* and 29 *GG*) of the subjects using Sanger sequencing and confirmed a concordance rate of 100% between array and sequencing data.

After a regional imputation and fine mapping for the rs7300146 region, 8 variants exceeding the genome-wide significance threshold were identified (Supplementary Table S1). Among them, rs122989222 (p = 6.16 × 10^−9^, OR = 3.02) showed the most significant association and the rs12309809 (p = 8.96 × 10^−8^, OR = 2.93) was associated with the cis expression quantitative trait locus (eQTL) for *SLC15A4*, which has been known an SLE susceptibility gene in a Chinese population.

### Predictive model for cSLE using an ROC curve analysis

**Figure 3:**
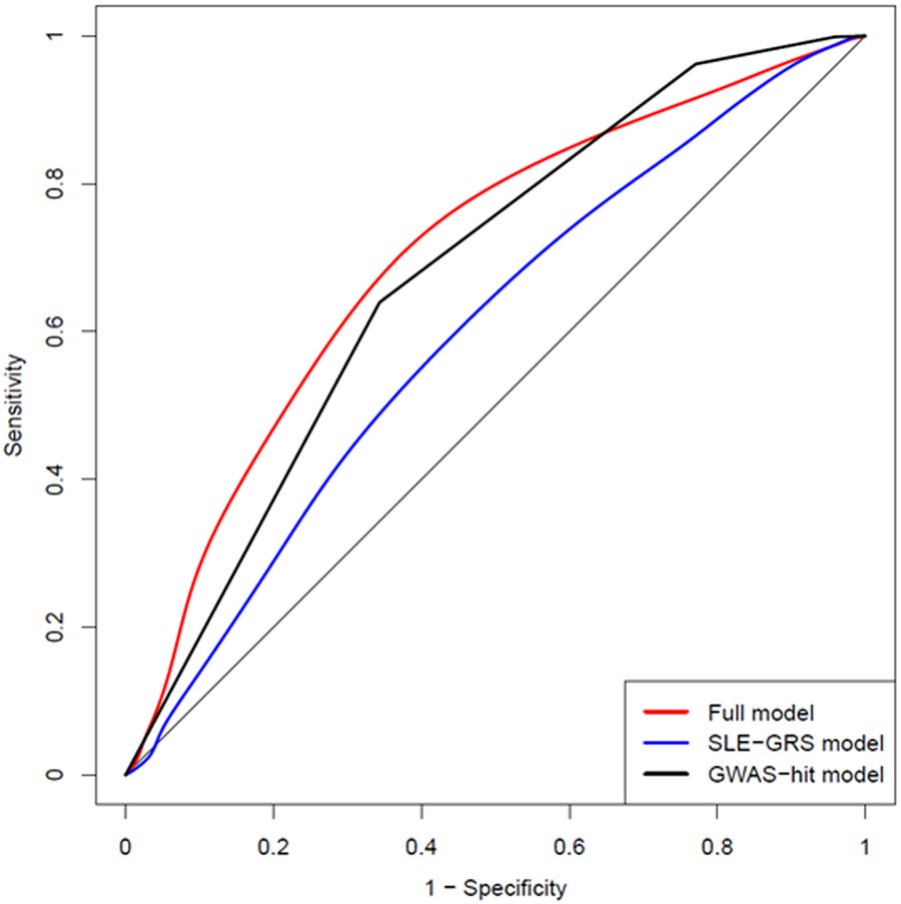
ROC curves from cSLE-prediction models including the weighted GRS for SLE risk (blue – SLE-GRS model with AUC = 0.60), the two novel cSLE-risk variants in the GWAS (black – GWAS-hit model with AUC = 0.68) or both the GRS and GWAS variants (red – full model with AUC = 0.71). ROC; receiver operating characteristics, SLE; systemic lupus erythematosus, GRS; genetic risk score, AUC; area under the curve, GWAS; genome-wide association study.

We further estimated the predition efficacy of the model constructed with weighted SLE GRS and two novel cSLE-risk loci. These predictors were added seperately or together into a multivariate model and AUCs of the ROC curve were calculated. The model with the weighted SLE GRS showed a ROC curve with an AUC of 0.6 and the model with the two cSLE-risk SNPs (rs7460469 and rs7300146) showed an AUC of 0.68, bringing the full model combined with the separate models to the highest predictive value with the AUC of 0.71 (Fig. 3).

## Discussion

We have demonstrated that cSLE known as worse phenotype than aSLE is associated with a higher accumulation SLE-risk effect, which might explain worse clinical feature in cSLE. In addition, for the first time, we newly identified two genes associated cSLE susceptibility, *XKR6* and *GLT1D1*. There findings allowed us to construct a single model to predict cSLE.

Webb *et al*. demonstrated that cSLE (<18 years at onset) was associated with the increased number of SLE-risk variants than aSLE^4^. They counted 19 SLE-risk alleles in each individual and found the significant difference between cSLE and aSLE in Gullah and African-American patients with SLE, but not Hispanic and European-American patients with SLE. Asian patients with SLE were excluded due to small sample size. In our study, we showed that SLE patients with higher GRS were significantly enriched in the cSLE than aSLE group. In contrast to Webb *et al*. study, we used large-size Korean cohort and calculated GRS from the most updated, well-validated Asian SLE-risk loci and HLA-DRβ1 amino-acid haplotype model. The weighted GRS used in the study was calculated based on the odds ratio as well as the total number of risk-variant copies.

In addition, our study revealed the genetic contribution on the age of SLE onset at individual variants within the two loci. First, *GLT1D1* around rs7300146 has been known as a candidate oncogene of colorectal cancer and relapse marker of multiple myeloma^9,10^. The SNP rs12309809, the proxy SNP of rs7300146 in *GLT1D1* was associated with expression level of the neighboring gene *SLC15A4*. It is known that *SLC15A4* is indispensable to innate immunity and antibody isotype switching to IgG2c^11,12^. Lack of *SLC15A4* is related with impaired function of SLE pathogenesis associated cytokines and protein, such as toll-like receptor 7 (TLR7)- and TLR9 dependent cytokines including type 1 interferon (IFN) and nucleotide oligomerization domain-1 (NOD1)^13,14^. Interestingly, the genetic variants in *SLC15A4* was associated with SLE risk in a Chinese population^15^ and lupus nephritis in a Caucasian population^16^.

Another new locus associated with cSLE was *XKR6*, which is located between *FAM167A* and *BLK*. This locus was associated with lupus nephritis as well as SLE susceptibility in multiple ancestreis^5–8,17^. However, the cSLE-associated SNP in *XKR6* that we identified in the study is Asian-specific and has no correlation with the SLE-risk, functional SNPs reported in previous studies^5^. Furthermore, the associated SNP in *XKR6* had no proxy SNPs in the flanking region and no functional effects reported so far. Thus, it may need more studies on this region to understand biological importance.

Consistent with reported observations^2,18,19^, lupus nephritis was more common in cSLE (60.4%) than aSLE (46.4%) in the study cohort (p = 0.010). Although the regions around the two novel cSLE-risk variants have been associated with lupus nephritis^16,17^, we note that the associations of the two newly identified variants were not explained by lupus nephritis. There was no association between lupus nephritis and the two cSLE-risk variants (p > 0.05 in 376 lupus nephritis-positive SLE vs. 405 lupus nephritis-negative SLE).

The limitation of the study is the relatively small sample size (n = 781) and lack of replication in independent cohorts. However, we could use the samples with high-density genotyping data and accurate clinical information including onset age which was gathered in the prospective Hanyang BAE Lupus cohort.

In conclusion, we reported the presence of genetic effects on the age of SLE onset, especially in the SLE susceptibility loci and the two novel cSLE loci, providing the first model to predict cSLE in Koreans.

## Methods

### Ethic statement

This study protocol was approved by the Institutional Ethics Review Board of Hanyang University Hospital and written informed consent was obtained from all participants in accordance with the principles of the Helsinki Declaration. All methods were carried out in accordance with the relevant guidelines and regulations.

### Study population

A total of 781 Korean patients with SLE were selected from the Hanyang BAE Lupus cohort^20^. cSLE (n = 96) was defined as SLE patients with onset age of below 16 and aSLE (n = 685) was defined as those with onset age of 16 or above^2^. As it is well known that hormones such as estrogen influence the development of SLE and flares, we restrict cSLE to patients below 16 based their stage of puberty, in which stage 5 of puberty is defined as the stage after 16 years when height no longer increases.

### Genotype data

All the study subjects were previously genotyped by both Immunochip and HumanOmniExpress genome-wide arrays, as previously described^21,22^. In brief, the merged array data was passed the general quality control thresholds such as call rates per individual or SNP (>95%), HWE in autosomal SNPs (p < 1 × 10^−5^), and minor allele frequency (MAF > 0.01). Principal component (PC) analysis calculated PCs to adjust for population stratification among the subjects in the subsequent statistical models. We noted that there were no genetic outliers of >6 standard deviations for each of the top 10 PCs. Ungenotyped variants in the associated loci were imputed by IMPUTE2 and passed the imputation quality score (info) of 0.6.

### Genetic risk score for SLE risk

Individual’s weighted GRS from well-validated SLE susceptibility loci were calculated to measure the accumulation effect of SLE susceptibility loci on the age of SLE onset. The SLE susceptibility loci used in calculation of GRS were composed with 45 Asian confirmed non-HLA SNPs from Sun *et al*. study^21^ and HLA-DRB1 haplotypes (constructed from HLA-DRβ1 amino-acid positions 11, 13, and 26) in our previous study^23^. We weighted the number of effect alleles by the previously reported effect size (=the natural logarithm of the previously reported odds ratio) for both non-HLA and HLA loci, and then summed all the locus-specific weighted score, as previously described^24^.

### Statistical analysis

The associations of weighted GRS with the age of SLE onset and the development of cSLE were tested using a linear regression and a logistic regression adjusting for the top 10 PCs as covariates, respectively. The association of each of single variants in the whole genome with cSLE were evaluated by a multivariable logistic regression analysis controlling for the top 10 PCs to calculate an odds ratio (OR) and its 95% confidence interval (95% CI) of the minor allele. Then, we estimated the predictive power of weighted GRS and/or risk loci of cSLE by measuring the area under the curve (AUC) of receiver operating characteristics (ROC). All data were analyzed with using the PLINK and R.

## Electronic supplementary material

Supplementary table 1

## References

[1] Mina R, Brunner HI (2013). Update on differences between childhood-onset and adult-onset systemic lupus erythematosus. Arthritis Res Ther.

[2] Joo YB, Park SY, Won S, Bae SC (2016). Differences in Clinical Features and Mortality between Childhood-onset and Adult-onset Systemic Lupus Erythematosus: A Prospective Single-center Study. J Rheumatol.

[3] Taylor KE (2011). Risk alleles for systemic lupus erythematosus in a large case-control collection and associations with clinical subphenotypes. PLoS Genet.

[4] Webb R (2011). Early disease onset is predicted by a higher genetic risk for lupus and is associated with a more severe phenotype in lupus patients. Ann Rheum Dis.

[5] Guthridge JM (2014). Two functional lupus-associated BLK promoter variants control cell-type- and developmental-stage-specific transcription. Am J Hum Genet.

[6] Harley IT, Kaufman KM, Langefeld CD, Harley JB, Kelly JA (2009). Genetic susceptibility to SLE: new insights from fine mapping and genome-wide association studies. Nat Rev Genet.

[7] Lee HS (2014). Ethnic specificity of lupus-associated loci identified in a genome-wide association study in Korean women. Ann Rheum Dis.

[8] Zhou, Y., Li, X., Wang, G. & Li, X. Association of FAM167A-BLK rs2736340 Polymorphism with Susceptibility to Autoimmune Diseases: A Meta-Analysis. *Immunol Invest* **45**, 336-348 (2016). 10.3109/08820139.2016.1157812 27105348

[9] Gylfe AE (2013). Identification of candidate oncogenes in human colorectal cancers with microsatellite instability. Gastroenterology.

[10] Krzeminski P (2016). Integrative analysis of DNA copy number, DNA methylation and gene expression in multiple myeloma reveals alterations related to relapse. Oncotarget.

[11] Lee J (2009). pH-dependent internalization of muramyl peptides from early endosomes enables Nod1 and Nod2 signaling. J Biol Chem.

[12] Dosenovic P (2015). Slc15a4 function is required for intact class switch recombination to IgG2c in response to TLR9 stimulation. Immunol Cell Biol.

[13] Sasawatari S (2011). The solute carrier family 15A4 regulates TLR9 and NOD1 functions in the innate immune system and promotes colitis in mice. Gastroenterology.

[14] Nakamura N (2014). Endosomes are specialized platforms for bacterial sensing and NOD2 signalling. Nature.

[15] Han JW (2009). Genome-wide association study in a Chinese Han population identifies nine new susceptibility loci for systemic lupus erythematosus. Nat Genet.

[16] Wang C (2013). Genes identified in Asian SLE GWASs are also associated with SLE in Caucasian populations. Eur J Hum Genet.

[17] Alonso-Perez E (2012). Further evidence of subphenotype association with systemic lupus erythematosus susceptibility loci: a European cases only study. PLoS One.

[18] Papadimitraki ED, Isenberg DA (2009). Childhood- and adult-onset lupus: an update of similarities and differences. Expert Rev Clin Immunol.

[19] Livingston B, Bonner A, Pope J (2011). Differences in clinical manifestations between childhood-onset lupus and adult-onset lupus: a meta-analysis. Lupus.

[20] Joo YB, Bae SC (2015). Assessment of clinical manifestations, disease activity and organ damage in 996 Korean patients with systemic lupus erythematosus: comparison with other Asian populations. Int J Rheum Dis.

[21] Sun C (2016). High-density genotyping of immune-related loci identifies new SLE risk variants in individuals with Asian ancestry. Nat Genet.

[22] Lessard CJ (2016). Identification of a Systemic Lupus Erythematosus Risk Locus Spanning ATG16L2, FCHSD2, and P2RY2 in Koreans. Arthritis Rheumatol.

[23] Kim K (2014). The HLA-DRbeta1 amino acid positions 11-13-26 explain the majority of SLE-MHC associations. Nat Commun.

[24] Kim K (2015). Interactions between amino acid-defined major histocompatibility complex class II variants and smoking in seropositive rheumatoid arthritis. Arthritis Rheumatol.

