# Publisher Correction: Genetic variants in systemic lupus erythematosus susceptibility loci, *XKR6* and *GLT1D1* are associated with childhood-onset SLE in a Korean cohort

**DOI:** 10.1038/s41598-018-30082-9

**Published:** 2018-07-31

**Authors:** Young Bin Joo, Jiwoo Lim, Betty P. Tsao, Swapan K. Nath, Kwangwoo Kim, Sang-Cheol Bae

**Affiliations:** 1 Department of Rheumatology, St. Vincent’s Hospital, The Catholic University of Korea, Suwon, Republic of Korea; 2 Department of Biology, Kyung Hee University, Seoul, Republic of Korea; 3 Division of Rheumatology and Immunology, Department of Medicine, Medical University of South Carolina, Charleston, South Carolina USA; 4 Arthritis and Clinical Immunology Research Program, Oklahoma Medical Research Foundation, Oklahoma City, Oklahoma USA; 5 Department of Rheumatology, Hanyang University Hospital for Rheumatic Diseases, Seoul, Republic of Korea

Correction to: *Scientific Reports* 10.1038/s41598-018-28128-z, published online 02 July 2018

In the original version of this Article, the author Young Bin Joo was incorrectly indexed. This error has now been corrected.

