# Supplementary material for: Genetic variants in systemic lupus erythematosus susceptibility loci, XKR6 and GLT1D1 are associated with childhood-onset SLE in a Korean cohort
Source: Sci Rep. 2018 Jul 2;8:9962. doi: 10.1038/s41598-018-28128-z (PMC6028392; doi:10.1038/s41598-018-28128-z)
Supplement: Supplementary file 1 — Supplementary table 1 [file 41598_2018_28128_MOESM1_ESM.docx]

**Genetic variants in systemic lupus erythematosus susceptibility loci, *XKR6* and *GLT1D1* are associated with childhood-onset SLE in a Korean cohort**

Young Bin Joo^1^, Jiwoo Lim^2^, Betty P. Tsao^3^, Swapan K. Nath^4^, Kwangwoo Kim^2*^, Sang-Cheol Bae^5*^

**Author affiliations**

^1^Department of Rheumatology, St. Vincent's Hospital, The Catholic University of Korea, Suwon, Republic of Korea

^2^Department of Biology, Kyung Hee University, Seoul, Republic of Korea

^3^Division of Rheumatology and Immunology, Department of Medicine, Medical University of South Carolina, Charleston, South Carolina, USA

^4^Arthritis and Clinical Immunology Research Program, Oklahoma Medical Research Foundation, Oklahoma City, Oklahoma, USA

^5^Department of Rheumatology, Hanyang University Hospital for Rheumatic Diseases, Seoul, Republic of Korea

Supplementary table S1. Significant associations of eight variants in the *GLT1D1* region with childhood-onset SLE (p<5x10^-8^)

| Chr | SNP | Major allele | Minor allele | MAF  in aSLE | MAF  in cSLE | OR for  minor allele | 95% CI | p | Impute2’s INFO score |
| --- | --- | --- | --- | --- | --- | --- | --- | --- | --- |
| 12 | rs12298922* | C | T | 0.166 | 0.346 | 3.02 | 2.08-4.38 | 6.16 x 10^-9^ | 0.97 |
| 12 | rs12309809* | G | A | 0.169 | 0.347 | 2.93 | 2.03-4.23 | 8.96 x 10^-9^ | 0.98 |
| 12 | rs12320015* | C | T | 0.168 | 0.342 | 2.97 | 2.05-4.32 | 1.06 x 10^-8^ | 0.97 |
| 12 | rs11059996* | C | G | 0.168 | 0.342 | 2.96 | 2.04-4.30 | 1.15 x 10^-8^ | 0.97 |
| 12 | rs7300146 | A | C | 0.173 | 0.349 | 2.85 | 1.98-4.10 | 1.49 x 10^-8^ | 1.00 |
| 12 | rs56266065* | G | A | 0.167 | 0.340 | 2.93 | 2.02-4.26 | 1.74 x 10^-8^ | 0.97 |
| 12 | rs61489254* | G | T | 0.162 | 0.337 | 2.93 | 2.01-4.27 | 2.02 x 10^-8^ | 0.95 |
| 12 | rs75130933* | C | CAG | 0.166 | 0.339 | 2.92 | 2.01-4.25 | 2.19 x 10^-8^ | 0.97 |

* Imputed

OR; odds ratio, CI; confidence interval
